# Clinical and regional distribution of TTN variants in toe walking: A descriptive cohort study

**DOI:** 10.1016/j.gmg.2026.100112

**Published:** 2026-06-01

**Authors:** David Pomarino, Amel Sidi Athmane, Bastian Fregien, Alexander Nazarkin, Kevin M. Rostásy

**Affiliations:** a Pomarino Praxis für Ganganomalien, Hamburg, Germany; b ORTHOMAX, orthopädische üBAG GbR Langenhagen, Germany; c Fakultät für Gesundheit (Department für Humanmedizin), Universität Witten/Herdecke, Herdecke, Witten, Germany

**Keywords:** Genetic Testing, Toe Walking, Neurology, Pediatrics, TTN

## Abstract

**Background:**

The TTN gene encodes titin, a key structural protein of the sarcomere. Variants in TTN are frequently identified in genetic testing, but their clinical interpretation remains challenging due to their high prevalence in the general population and frequent classification as variants of uncertain significance (VUS). Toe walking is often considered idiopathic; however, emerging evidence suggests a potential genetic contribution.

**Objective:**

To provide a descriptive analysis of TTN variants in children presenting with persistent toe walking, using a region-based approach to explore variant distribution and associated clinical features.

**Methods:**

This retrospective study included children with persistent toe walking following exclusion of known neurological, neuromuscular, and orthopedic causes. All patients underwent standardized clinical assessment and targeted next-generation sequencing using a 49-gene panel. TTN variants were grouped into predefined cDNA intervals (c.1–c.9000). Clinical and genetic data were analyzed descriptively.

**Results:**

Fifty-five patients carrying 43 TTN variants were included (mean age 7.3 years; 71% male). Variants were unevenly distributed, with clustering in the c.1001–2000 (22.4%) and c.3001–4000 (19.0%) regions. All variants were classified as VUS. A consistent clinical phenotype was observed across groups, including persistent toe walking, reduced ankle dorsiflexion, and a frequent positive foot drop test. Group-specific differences were noted but remained inconsistent and descriptive.

**Conclusions:**

TTN variants in children with toe walking show heterogeneous distribution but a largely consistent clinical presentation. No clear genotype–phenotype correlation was identified. These findings highlight the need for cautious interpretation of TTN VUS and further studies to clarify their clinical significance.

## Introduction

Titin, encoded by the TTN gene, is the largest known human protein and plays a central role in the structural and functional organization of the sarcomere. Extending from the Z-disk to the M-line, titin contributes to passive elasticity, mechanical stability, and intracellular signaling in striated muscle. [1], [2], [3], [4] Due to its exceptional size and complex architecture, TTN contains a large number of genetic variants, many of which are classified as variants of uncertain significance (VUS).

TTN variants have been associated with a wide spectrum of cardiac and skeletal muscle disorders. [5] However, interpretation of these variants remains challenging, particularly in individuals with mild or nonspecific clinical findings, as TTN variation is also common in the general population. As a result, establishing clear genotype–phenotype relationships is often difficult. [6]

Interpretation of TTN variants remains particularly difficult because TTN exhibits substantial background variation in population databases, and variant pathogenicity may depend on multiple factors including exon usage, protein domain localization, transcript expression, and inheritance patterns. Consequently, simple identification of a TTN variant is insufficient to infer clinical relevance.

In clinical practice, TTN variants are increasingly identified in patients presenting with gait abnormalities, including toe walking. Toe walking has traditionally been described as idiopathic; however, increasing evidence suggests that genetic factors, including TTN variation, may contribute to its manifestation. Current evidence remains largely descriptive, and a definitive causal relationship between specific TTN variants and isolated toe walking has yet to be established. [7], [8], [9]

Given the size of the TTN gene, analyzing variants as a single group may obscure potential distribution patterns. A region-based approach may provide a clearer descriptive overview of variant localization and associated clinical features.

Although TTN has been extensively studied in cardiomyopathies and neuromuscular disorders, limited data exist regarding the distribution of TTN variants in children presenting primarily with persistent toe walking or related gait abnormalities. Furthermore, few studies have explored whether descriptive clustering of TTN variants across predefined genomic regions may reveal recognizable phenotypic patterns.

The aim of this study was to describe the distribution of TTN variants identified in a clinically characterized cohort of children with persistent toe walking and to explore whether descriptive differences in associated clinical features emerge across predefined cDNA intervals.

Due to the large size of the TTN gene, variants were grouped into predefined cDNA intervals to enable a structured analysis of their distribution. The present study focuses on variants within the c.1–c.9000 region and describes their associated clinical characteristics.

This work represents part of a broader research project investigating TTN variant distribution across the gene. The current analysis is descriptive in nature and does not aim to establish causality, but rather to identify patterns that may inform future studies.

## Methods

### Study Design and Cohort

This study represents a retrospective descriptive analysis of children evaluated for persistent toe walking at a specialized referral center. The study population was drawn from approximately 2000 patients undergoing standardized clinical assessment.

Patients assessed between November 2022 and April 2026 were screened for eligibility. Clinical and genetic data were analyzed for all patients meeting predefined inclusion criteria at the time of database closure.

The study was conducted in accordance with the ethical principles of the World Medical Association Declaration of Helsinki. All procedures complied with applicable legal and institutional requirements and were approved by the ethical board of the Deutschen Verbandes für Physiotherapie at the Physio-Akademie in Wremen, Germany (project number 2025–02).

Inclusion in the genetic analysis was based on a clinical diagnosis of persistent toe walking following structured diagnostic evaluation. All included patients underwent genetic testing as part of routine clinical work-up, without prior knowledge of specific gene status.

Patients were referred through established clinical pathways, either directly for primary evaluation or following prior assessment in specialized centers. Inclusion required the absence of a defined neurological, neuromuscular, or orthopedic cause of toe walking after comprehensive evaluation.

Children with confirmed alternative diagnoses (e.g., cerebral palsy, autism spectrum disorder, muscular dystrophy, tethered cord syndrome) or structural deformities unrelated to neuropathic mechanisms were excluded.

**Fig. 1:**
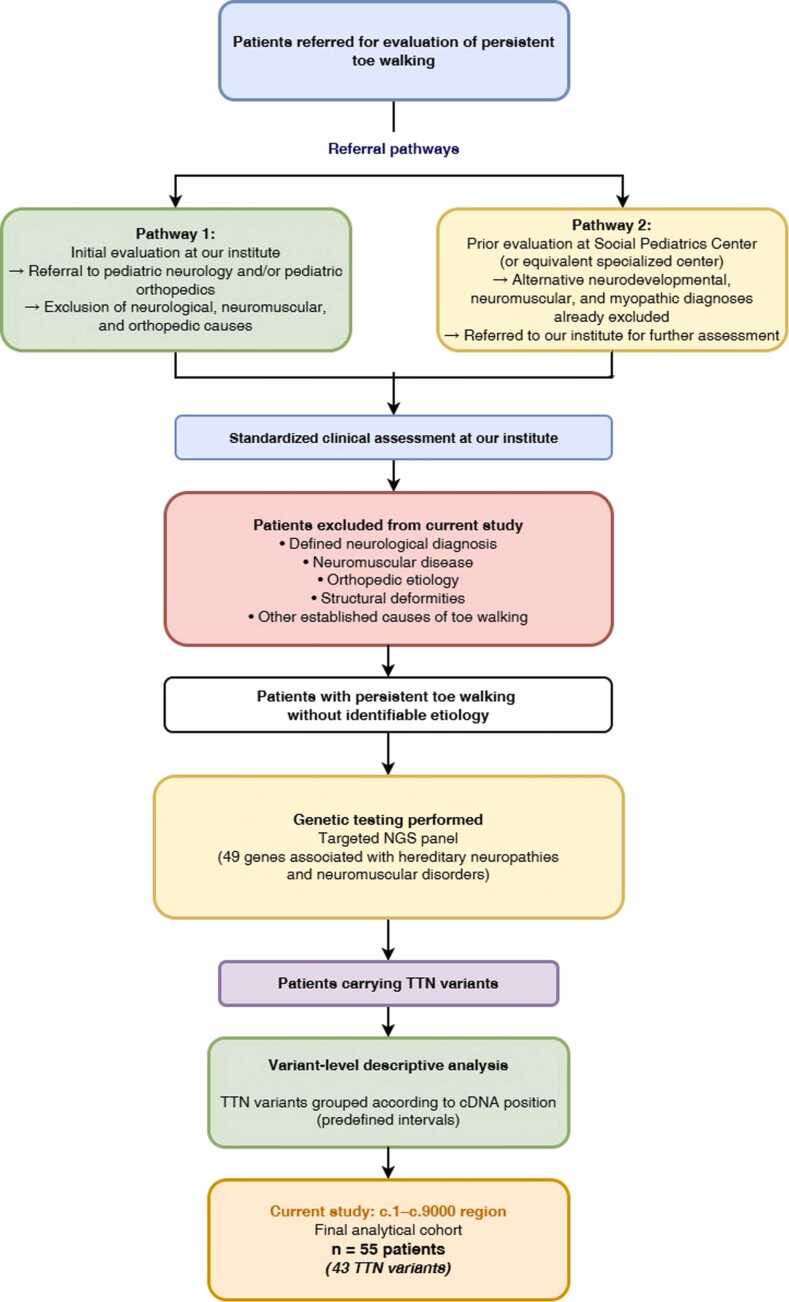
Patient selection and analytical workflow.

A schematic overview of referral pathways, clinical assessment, eligibility determination, genetic testing, and selection of the final analytical cohort is presented in Fig. 1.

### Clinical Examination and Assessment Procedures

All children underwent a standardized clinical evaluation that was performed independently and blinded to the results of genetic testing. The assessment followed a predefined protocol including detailed history taking, musculoskeletal and postural assessment, gait evaluation, and neurological examination, as outlined below.

1. Patient History

A structured medical history was obtained from parents or primary caregivers. The following information was systematically collected:

• Demographic data (age, sex), age at independent walking, and age at onset of toe walking
• Pattern and progression of the gait abnormality
• Family history of toe walking or hereditary neuropathies
• Presence of muscular symptoms, including muscle pain, cramps, or weakness
• Developmental history, including speech or language delay and social or behavioral abnormalities
• Presence of visual impairment

## Physical Examination

### Postural and Structural Assessment

A comprehensive morphological evaluation was conducted to identify structural abnormalities. The following features were assessed:

• Lumbar hyperlordosis
• Pes cavus
• Foot drop
• Thoracic deformities, including pectus excavatum and pectus carinatum
• Digital anomalies, specifically clinodactyly and brachydactyly

### Balance and Motor Function

Balance was assessed using a standardized single-leg stance test:

• Inability to maintain a single-leg stance for ≥ 5 s without support was considered abnormal
  Motor function was evaluated using functional standing tasks:
• Heel walking test: inability to maintain heel walking was interpreted as weakness of ankle dorsiflexors
• Foot drop test (active toe-lift): inability to lift the forefoot against gravity was considered indicative of dorsiflexor weakness or impaired motor control

### Ankle Range of Motion (ROM)

Ankle range of motion was measured using a goniometer according to the neutral (0°) method²:

• Dorsiflexion: considered restricted if < 20°
• Plantarflexion: considered restricted if < 50°

Measurements were performed in the supine position with knees extended to differentiate between gastrocnemius and soleus muscle contributions.

### Neurological Examination

Neurological assessment included:

• Deep tendon reflexes (patellar):
  The patellar reflex was elicited with the patient in a seated position and legs relaxed. The patellar tendon was tapped using a reflex hammer, with a normal response defined as a brief knee extension³ . Reduced or absent responses were recorded as hyporeflexia or areflexia.
• Tremor assessment:

Patients were examined with arms extended forward and palms facing downward. Repetitive opening and closing of the hands was used as a provocation maneuver. Tremor was considered present if rhythmic oscillatory movements occurred during or after the task.

### Genetic Analysis

Genetic testing was performed using a targeted next-generation sequencing (NGS) approach designed to detect variants associated with hereditary neuropathies and myopathies that may present with gait abnormalities. Only patients analyzed using this standardized NGS workflow were included; individuals tested using alternative methods (e.g., whole-exome sequencing) were excluded to ensure methodological consistency.

All samples were processed using a validated 49-gene panel (Thermo Fisher Scientific), comprising genes implicated in neuromuscular and neuropathic disorders (Supplementary Table 1). Panel design was guided by Human Phenotype Ontology (HPO) terms related to neuro- and myopathies.

Saliva samples were collected during routine clinical evaluation. DNA extraction followed standard procedures, and sequencing was performed on the Ion Torrent platform (Thermo Fisher Scientific, Waltham, MA, USA). Target regions included all coding exons and adjacent intronic boundaries (±10 bp).

Copy-number variation (CNV) analysis was incorporated into the workflow. Suspected deletions or duplications identified during NGS were confirmed using Multiplex Ligation-dependent Probe Amplification (MLPA) with PMP22-specific probes (MRC-Holland), enabling detection of clinically relevant CNVs, including the PMP22 duplication associated with CMT1A.

Raw sequence data were analyzed using SeqPilot SeqNext software (version 5.3.3). Variants were considered for interpretation only if sequencing depth was ≥ 30 × and allele read frequency exceeded 20%.

Variant classification followed ACMG guidelines, categorizing variants as pathogenic, likely pathogenic, variant of uncertain significance (VUS), likely benign, or benign. All classifications were time-stamped to reflect updates in population databases and annotation tools. Only pathogenic, likely pathogenic, and VUS variants were included in the analysis.

Variant interpretation was supported by in silico prediction tools (e.g., MutationTaster, PolyPhen−2, Mutation Assessor) and multiple databases, including HGMD Professional, LOVD-IARC, dbSNP, and ClinVar.

The applied sequencing approach does not fully exclude variants outside the targeted regions, such as deep intronic or regulatory variants, and may have limited sensitivity for low-level mosaicism or single-exon CNVs.

According to validation data from the accredited diagnostic laboratory (Labor Dr. Heidrich & Colleagues, Hamburg, Germany), analytical sensitivity for clinically relevant variants exceeded 96%. Documentation of validation procedures is available upon request. Segregation analysis was not routinely performed due to feasibility and cost considerations.

### Variant Grouping Strategy

Given the large size and structural complexity of the TTN gene, a variant-based analytical approach was applied.

Variants were grouped according to their cDNA position into predefined intervals (e.g., c.1001–2000, c.2001–3000, etc.) to enable region-by-region comparison across the gene.

Each variant was treated as an independent observation; therefore, patients carrying multiple variants could contribute to more than one group.

The flowchart was created using diagrams.net (draw.io), version 26.2.2

### Statistical Analysis

Descriptive statistical methods were applied to characterize demographic variables, clinical findings, and genetic results.

Analyses were performed using LibreOffice Calc (version 25.2.5.2).

Categorical variables were summarized as counts and percentages. Percentages were calculated using available-case denominators with missing values excluded.

Continuous variables were summarized using mean values and standard deviations where applicable.

Exact 95% confidence intervals for proportions were estimated using Fisher’s exact method where appropriate.

No formal hypothesis testing, multivariable modeling, correction for multiple testing, or inferential genotype–phenotype analyses were performed because of the exploratory and descriptive study design.

## Results

### Cohort overview

A total of 55 patients carrying 43 TTN variants were included in the analysis. The cohort comprised 39 males and 16 females, with a mean age of 7.3 years.

### Variant distribution

**Fig. 2:**
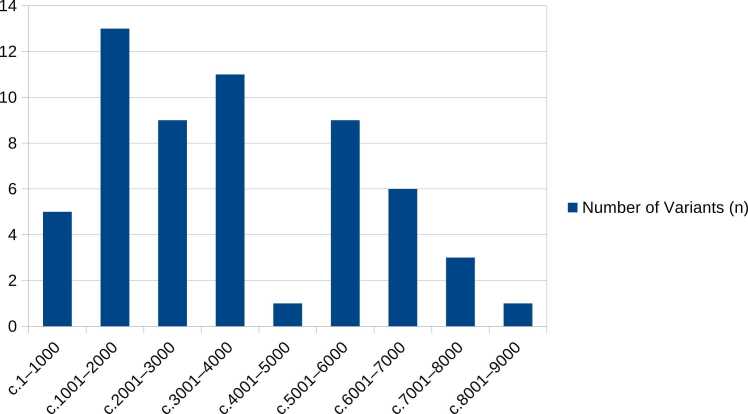
Distribution of TTN variants across cDNA intervals.

TTN variants were unevenly distributed across the analyzed cDNA intervals, with the highest frequencies observed in the c.1001–2000 and c.3001–4000 regions (Table 1). The distribution of variants across cDNA intervals is further illustrated in Fig. 2.

**Table 1:** Variant distribution.

cDNA Interval	Number of Variants (n)	Percentage (%)
c.1–1000	5	8.6%
c.1001–2000	13	22.4%
c.2001–3000	9	15.5%
c.3001–4000	11	19.0%
c.4001–5000	1	1.7%
c.5001–6000	9	15.5%
c.6001–7000	6	10.3%
c.7001–8000	3	5.2%
c.8001–9000	1	1.7%

### Variant characteristics

All of the variants were classified as variants of uncertain significance (VUS), with no pathogenic variants identified.

### Core Clinical Features Across All Groups

The main clinical characteristics shared across variant groups are summarized in Table 2.

**Table 2:** Core features.

Feature	Observation
Reduced ankle mobility	Very frequent (80–100%)
Positive drop foot test	Nearly universal
Age at walking	Normal (∼12–14 months)
Gender	Predominantly Male
Family history	Frequent (≈30–100% depending on group)
Clino/brachydactyly	Highly prevalent (∼70–100%)

### Group comparison

Variant-related findings across the defined cDNA intervals are summarized in Supplementary Table 1. Analyses were performed at the variant level; therefore, some variants were identified in more than one patient, and individual patients could carry multiple variants across different intervals. Accordingly, values represent variant counts rather than unique patient numbers. Detailed clinical characteristics across cDNA-defined groups are provided in Supplementary Table 1. A separate Supplementary Table 2 contains a comprehensive overview of all detected variants, including patients harboring more than one TTN variant as well as variants identified in other genes included in the 49-gene panel.

Based on this grouping, clinical features were compared descriptively across cDNA-defined intervals.

The largest variant groups (c.1001–2000, c.2001–3000, c.3001–4000, and c.5001–6000) were analyzed in detail, as they accounted for the majority of identified variants.

Groups with very small sample sizes (n ≤ 3) were not analyzed individually due to limited interpretability.

The c.1001–2000 group, representing the largest number of variants, demonstrated a broader clinical spectrum, including a higher frequency of speech or language delay, prominent muscle pain, and the presence of spinal abnormalities.

The c.3001–4000 group was characterized predominantly by musculoskeletal features, with muscle-related symptoms representing the main clinical finding.

The c.2001–3000 group showed a high prevalence of structural features, particularly clinodactyly/brachydactyly and pectus carinatum.

The c.5001–6000 group demonstrated a mixed phenotype, including frequent muscle pain, as well as higher frequencies of visual impairment and pes cavus. In addition, tremor was observed in this group.

The c.1–1000 and c.6001–7000 groups demonstrated clinical features comparable to those observed in the larger variant groups, without distinct or group-specific patterns. Core musculoskeletal findings, including reduced ankle dorsiflexion and frequent positive foot drop test, were consistently present.

## Discussion

This study provides a descriptive overview of TTN variants across defined cDNA intervals in patients presenting with persistent toe walking. Variants were unevenly distributed across the gene, with clustering observed in the c.1001–2000 and c.3001–4000 regions. Across groups, a consistent core phenotype was identified, characterized by frequent toe walking, reduced ankle dorsiflexion, and a high prevalence of positive foot drop test.

Across all variant groups, patients demonstrated a largely consistent musculoskeletal phenotype. Early motor development was generally preserved, while toe walking and reduced ankle mobility were prominent features. This consistency across cDNA intervals suggests that the observed clinical presentation may not be restricted to a specific region of the TTN gene.

Despite the overall phenotypic consistency, certain groups showed differences in associated clinical features. Variants within the c.1001–2000 interval were associated with a broader clinical spectrum, including speech or language delay and spinal abnormalities. In contrast, the c.3001–4000 group was predominantly characterized by musculoskeletal features. Structural findings such as clinodactyly/brachydactyly and pectus carinatum were more frequently observed in the c.2001–3000 group, while the c.5001–6000 interval demonstrated a mixed phenotype including visual impairment, pes cavus, and tremor. However, these observations remain descriptive and should be interpreted with caution.

No clear genotype–phenotype correlation could be established based on variant location alone. Although certain trends were observed, findings were not consistent across all groups and may be influenced by sample size and variant heterogeneity.

The large size and complex structure of the TTN gene, together with the high prevalence of variants in the general population, complicate interpretation of variant significance. A region-based analytical approach was therefore applied in this study to explore potential distribution patterns; however, the clinical relevance of such grouping remains to be clarified.

A major strength of this study lies in the relatively large and systematically characterized cohort, in which both clinical and genetic assessments were performed using standardized protocols. In addition, clinical examinations were conducted independently of genetic results, reducing the risk of observer bias in phenotype documentation.

Several limitations should be considered when interpreting the findings.

First, electrophysiological data were not routinely available. Nerve conduction studies could provide additional insight into the presence of subtle or subclinical neuropathic involvement.

Second, the interpretation of variants of uncertain significance (VUS) remains subject to change. As population databases and functional evidence continue to expand, some variants identified in this study may be reclassified over time.

Third, the study cohort is subject to referral bias, as all participants were evaluated due to persistent toe walking. Consequently, the findings may not reflect the full phenotypic spectrum of individuals carrying TTN variants in the general population.

Fourth, the absence of a control cohort or enrichment analysis limits the ability to assess whether TTN variants are overrepresented in this population compared with background frequencies. Therefore, the observed association between TTN variants and toe walking may be incidental.

Fifth, segregation analysis was not performed. As a result, the inheritance pattern and phase (cis vs. trans) of multiple TTN variants identified in individual patients could not be determined. This limits the ability to draw conclusions regarding potential compound effects and restricts genotype–phenotype interpretation.

Finally, genetic testing was restricted to a predefined 49-gene panel targeting neuromuscular and neuropathic conditions associated with gait abnormalities. Variants in genes not included in this panel may therefore have been missed, representing a potential diagnostic limitation.

Further studies with larger cohorts and functional validation are required to clarify the role of TTN variants in patients with toe walking and to better define potential genotype–phenotype relationships.

## Funding Statement

This research did not receive any specific grant from any funding agency in the public, commercial, or not-for-profit sectors.

## Ethical statement

This study was conducted in accordance with the ethical principles of the World Medical Association Declaration of Helsinki. All procedures were performed in compliance with relevant laws and institutional guidelines and were approved by the ethical board of the

Deutschen Verbandes für Physiotherapie an der Physio-Akademie in Wremen, Germany (project number 2025–02).

Prior to participation, written informed consent was obtained from all subjects. The consent process included explanations of the study's purpose, procedures, and any potential implications of the results. All data collected were formally anonymized to protect participant confidentiality.

This manuscript was prepared in accordance with the International Committee of Medical Journal Editors (ICMJE) recommendations.

## CRediT authorship contribution statement

David Pomarino contributed to the conceptualization of the study, data curation, and formal analysis. Amel Sidi Athmane contributed to data curation and investigation and drafted the original manuscript. Bastian Fregien and Alexander Nazarkin contributed to the study methodology and validation. Kevin M. Rostásy contributed to formal analysis, supervision, validation, and manuscript review and editing. All authors reviewed and approved the final manuscript.

## Data availability

Raw next-generation sequencing data were generated as part of routine clinical diagnostics and are not publicly available because of ethical, privacy, and institutional restrictions governing human genetic data. De-identified variant-level data supporting the findings of this study are provided in Supplementary Table 2. Additional anonymized data may be made available from the corresponding author upon reasonable request and subject to ethical approval.

## Declaration of Generative AI and AI-assisted technologies in the writing process

Generative AI Deepseek was used in the writing phase of this manuscript exclusively for linguistic polishing and enhancing clarity. All scientific reasoning, data analysis, and intellectual substance remain the sole contribution of the authors. The AI was not employed in the research process itself.

## Declaration of Competing Interest

The Authors declare that there is no conflict of interest.

## Footnotes

Appendix A Supplementary data associated with this article can be found in the online version at doi:10.1016/j.gmg.2026.100112.
