# Supplementary material for: Clinical and regional distribution of TTN variants in toe walking: A descriptive cohort study
Source: Glob Med Genet. 2026 Jun 1;13(2):100112. doi: 10.1016/j.gmg.2026.100112 (PMC13276335; doi:10.1016/j.gmg.2026.100112)
Supplement: Supplementary file 2 — Supplementary material [file mmc2.docx]

Supplementary Table 2. Variant-level data for all patients, including TTN variants and additional variants identified in other genes from the targeted 49-gene panel.

| **Patient ID** | **TTN variant(s)** | **Other gene variant(s)** | **cDNA- groups** |
| --- | --- | --- | --- |
| **1** | **TTN  c.415C>T**  TTN c.47248G>A | ATM c.6820G>A  EGR2 c.1277G>A  KMT2C c.6339A>C  POLG c.2481-7C>T | **c.1 – 1000** |
| **2** | **TTN c.43G>A**  TTN c.55396G>A | ATM c.1229T>C  ATM c.5258A>G  DHTKD1 c.1079T>C  EPHB4 c.118G>A | **c.1 – 1000** |
| **3** | **TTN c.875C>T**  TTN c.12178G>A  TTN c.14450A>C  TTN c.34864G>A | CHRNE c.575T>C  COL6A3 c.3271G>A | **c.1 – 1000** |
| **4** | **TTN c.889C>T** | CREBBP c.5992G>A  PYGM c.160T>G | **c.1 – 1000** |
| **5** | **TTN c.910G>A** | — | **c.1 – 1000** |
| **6** | **TTN c.1137A>G** | CLCN1 c.2864A>T | **c.1001–2000** |
| **7** | **TTN c.1137A>G** TTN c.14327T>C  TTN c.98095C>T | COL6A2 c.1097G>A  MFN2 c.227T>G  PMP22  Duplication | **c.1001–2000** |
| **8** | **TTN c.1137A>G** | COL6A2 c.1562G>A  KMT2C c.4414C>T  SBF2 c.5020_5022delGAA | **c.1001–2000** |
| **9** | **TTN c.1137A>G** | — | **c.1001–2000** |
| **10** | **TTN c.1137A>G**  TTN c.42329T>C  TTN c.5993G>A | — | **c.1001–2000** |
| **11** | **TTN c.1137A>G** | — | **c.1001–2000** |
| **12** | **TTN c.1137A>G**  **TTN c.3608G>C**  TTN c.22439A>C  TTN c.3608G>C | RETREG1 c.607G>A | **c.1001–2000 c.2001–3000** |
| **13** | **TTN c.1137A>G**  **TTN c.5993G>A**  TTN c.106531+1G>A | ZFYVE26 c.2285G>A  ALS2 c.3513-8_3513-2delinsTTGCT  DHTKD1 c.1640T>C  NEFL c.581A>G | **c.1001–2000**  **c.5001–6000** |
| **14** | **TTN c.1141G>A**  TTN c.66601G>A | KMT2C c.1355A>G  PYGM c.645G>A | **c.1001–2000** |
| **15** | **TTN c.1213G>A**  TTN c.23338G>T | POLG c.1760C>T  POLG c.752C>T  PRX c.2548C>G  SBF1 c.4639C>T | **c.1001–2000** |
| **16** | **TTN c.1213G>A**  TTN c.23338G>T | PRX c.2548C>G  SBF1 c.4639C>T | **c.1001–2000** |
| **17** | **TTN c.1301A>G**  TTN c.26792A>T  TTN c.68458G>C  TTN c.73994C>T  TTN c.83618T>C | — | **c.1001–2000** |
| **18** | **TTN c.1800+1G>A** | — | **c.1001–2000** |
| **19** | **TTN c.2161G>A** | KMT2C c.9931C>T | **c.2001–3000** |
| **20** | **TTN c.2176T>C** | DHTKD1 c.923G>A  ZFYVE26 c.833C>T | **c.2001–3000** |
| **21** | **TTN c.2290G>A** | — | **c.2001–3000** |
| **22** | **TTN c.2354T>A**  TTN c.42023C>T | — | **c.2001–3000** |
| **23** | **TTN c.2423G>A** | ATXN3 c.916_917insAGCAGCAGCAGCAGCAGC  COL6A3 c.7425C>A  KMT2C c.4592C>T  SPTLC2 c.1114G>A | **c.2001–3000** |
| **24** | **TTN c.2536T>G**  TTN c.45408G>T  TTN c.52966G>A  TTN c.64457T>A  TTN c.64465C>T | ATM c.26T>A  CACNA1A c.5959A>G  GARS1 c.1429A>G  SBF1 c.1312C>T | **c.2001–3000** |
| **25** | **TTN c.2581G>C**  TTN c.78890C>T | COL6A3 c.1688A>G | **c.2001–3000** |
| **26** | **TTN c.2611G>T**  TTN c.11087A>G  TTN c.11672C>T  TTN c.21668G>A  TTN c.31757C>A  TTN c.72379G>A  TTN c.77813G>C  TTN c.88721G>A | IQSEC2 c.708-3C>T | **c.2001–3000** |
| **27** | **TTN c.2686G>A**  TTN c.104471A>G  TTN c.42353A>T | SBF1 c.1431C>T  SH3TC2 c.1000A>G  ZFYVE26 c.4301A>C | **c.2001–3000** |
| **28** | **TTN c.3002T>G**  TTN c.30485C>T  TTN c.62572A>G | — | **c.3001–4000** |
| **29** | **TTN  c.3100G>A**  TTN  c.91601A>T | — | **c.3001–4000** |
| **30** | **TTN c.3100G>A**  TTN c.69460A>G  TTN c.91601A>T | COL6A3 c.5610C>A | **c.3001–4000** |
| **31** | **TTN c.3190A>T**  TTN c.68067_68069delCAA | — | **c.3001–4000** |
| **32** | **TTN c.3241G>A** | — | **c.3001–4000** |
| **33** | **TTN c.3409G>C** | COL6A2 c.1306G>A | **c.3001–4000** |
| **34** | **TTN c.3409G>C** | TTN c.51712C>T | **c.3001–4000** |
| **35** | **TTN c.3409G>C** | — | **c.3001–4000** |
| **36** | **TTN c.3409G>C** | KMT2C c.6339A>C | **c.3001–4000** |
| **37** | **TTN c.3619C>A**  TTN c.31807G>A  TTN c.44077C>T  TTN c.7961G>A | PYGM c.1888G>A  TTR c.424G>A | **c.3001–4000** |
| **38** | **TTN c.4247G>A**  TTN c.11809A>C | ATXN7 c.1958C>T | **c.4001–5000** |
| **39** | **TTN  c.5200A>G** | — | **c.5001–6000** |
| **40** | **TTN c.5479G>T**  TTN c.81647G>A  TTN c.102271C>T | ATM c.3925G>A | **c.5001–6000** |
| **41** | **TTN c.5479G>T**  TTN c.102271C>T | OPA1 c.326T>C | **c.5001–6000** |
| **42** | **TTN c.5645G>A**  **TTN c.5698G>A** | EGR2 c.1154T>A  EPHB4 c.1905G>T  PRX c.1546C>T  SH3TC2 c.505T>C | **c.5001–6000** |
| **43** | **TTN c.5810C>G** | — | **c.5001–6000** |
| **44** | **TTN c.5810C>G**  TTN c.60626T>G | PYGM c.660G>A  SBF1 c.4927C>A | **c.5001–6000** |
| **45** | **TTN c.5810C>G** | CHRNE c.1019C>T  COL6A3 c.5734G>A | **c.5001–6000** |
| **46** | **TTN c.6304G>T**  TTN c.71222C>T | COL6A3 c.9061G>C | **c.6001–7000** |
| **47** | **TTN c.6353T>C**  TTN c.17818T>C | CACNA1A c.6650_6661delACCACCACCATC | **c.6001–7000** |
| **48** | **TTN c.6420T>A**  TTN c.60364G>A | — | **c.6001–7000** |
| **49** | **TTN c.6584A>G**  TTN c.81635T>C | COL6A3 c.5610C>A | **c.6001–7000** |
| **50** | **TTN c.6895T>C** | IQSEC2 c.809G>A | **c.6001–7000** |
| **51** | **TTN c.6913G>A** | COL6A2 c.791G>A  SBF1 c.4927C>A  ZFYVE26 c.6284A>G | **c.6001–7000** |
| **52** | **TTN c.7060C>T**  TTN c.31873C>G | — | **c.7001–8000** |
| **53** | **TTN c.7711G>A**  TTN c.23353T>C | — | **c.7001–8000** |
| **54** | **TTN c.7891G>A**  TTN c.34601T>C | — | **c.7001–8000** |
| **55** | **TTN c.8947A>G** | — | **c.8001–9000** |

**Genes analyzed in the present study are indicated in bold.*
